# Long-Term Memory Search across the Visual Brain

**DOI:** 10.1155/2012/392695

**Published:** 2012-07-19

**Authors:** Milan Fedurco

**Affiliations:** Michelin Recherche Technique, S.A., Route André-Piller 30, 1762 Givisiez, Fribourg, Switzerland

## Abstract

Signal transmission from the human retina to visual cortex and connectivity of visual brain areas are relatively well understood. How specific visual perceptions transform into corresponding long-term memories remains unknown. Here, I will review recent Blood Oxygenation Level-Dependent functional Magnetic Resonance Imaging (BOLD fMRI) in humans together with molecular biology studies (animal models) aiming to understand how the retinal image gets transformed into so-called visual (retinotropic) maps. The broken object paradigm has been chosen in order to illustrate the complexity of multisensory perception of simple objects subject to visual —rather than semantic— type of memory encoding. The author explores how amygdala projections to the visual cortex affect the memory formation and proposes the choice of experimental techniques needed to explain our massive visual memory capacity. Maintenance of the visual long-term memories is suggested to require recycling of GluR2-containing **α**-amino-3-hydroxy-5-methyl-4-isoxazolepropionic acid receptors (AMPAR) and **β**_2_-adrenoreceptors at the postsynaptic membrane, which critically depends on the catalytic activity of the N-ethylmaleimide-sensitive factor (NSF) and protein kinase PKM**ζ**.

## 1. Introduction

The McMillan Thesaurus dictionary [1] defines the brain as the organ inside your head that allows you to think, feel, and control your body. In addition to cognition, sensory, and motor systems, our brain handles the circadian sleep/wake cycle, body temperature, pain perception, and a myriad of other functions [2–7]. Their execution requires more than 86 billion neurons [8] connected in complex brain circuits [9–11]. Resulting neural networks rely on a fine balance between the excitatory and inhibitory neurons [12–14], operate in the subsecond range [15, 16], and manifest a high degree of plasticity at their synaptic contacts. The synaptic plasticity allows neurons not only to transmit the information, but also to learn, memorize, and retrieve the most important events [17].

**Figure 1:**
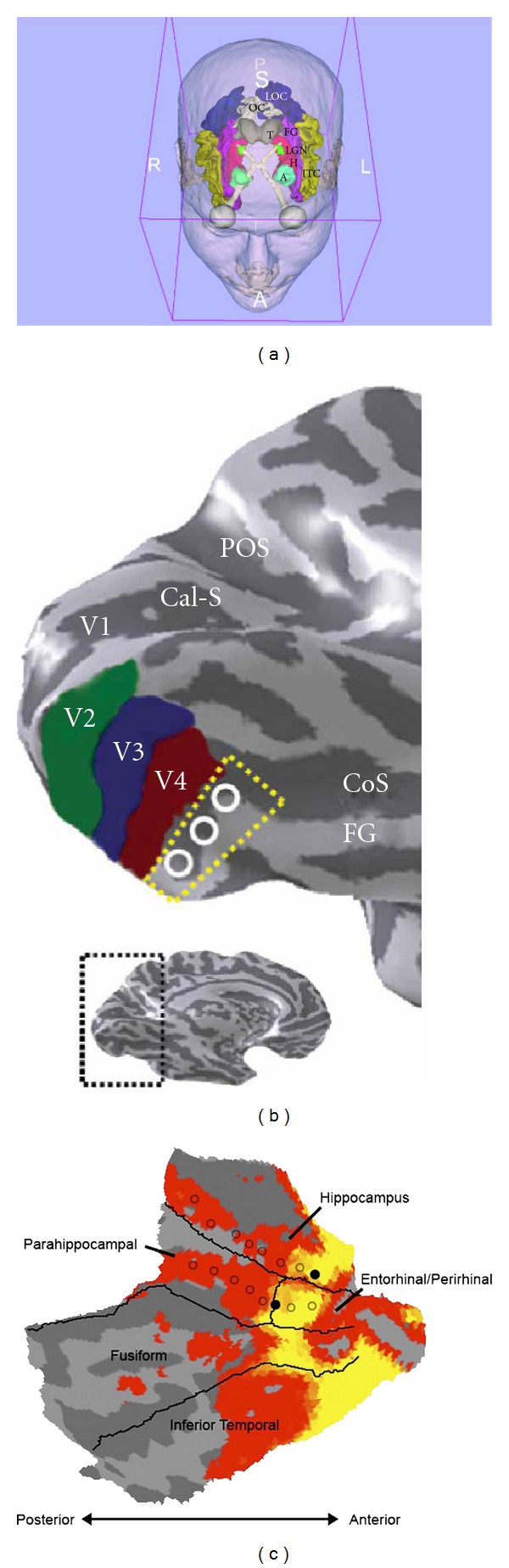
Parcellation of the human visual cortex and brain areas involved in visual object perception: (a) eye bulb—grey, optical tract/optical chiasm—white, lateral geniculate nucleus (LGN)—green, occipital visual cortex (OC)—white, lateral occipital gyrus (LOC)—navy blue, inferior temporal gyrus (ITC)—creamy yellow, fusiform gyrus (FG)—magenta, hippocampus (H)—red, amygdala (A)—cyan, thalamus (T)—grey (created using the 3D Brain Slicer [38]). (b) Ventral occipital visual field map models: Cal-S (V1)—calcarine sulcus, V2—green, V3—blue, V4 (brick red), FG—fusiform gyrus, CoS—collateral sulcus, POS—parietal occipital sulcus. The dotted yellow line and white circles denote the region of cortex in which the V4 and V8 models diverge (inset shows inflated model of the left human hemisphere). (c) Anterior part of the fusiform gyrus is located in the vicinity of the hippocampal formation. (b) reproduced, with permission, from [25] Macmillan Publishers Ltd. (c) (right) reproduced, with permission, from [39] the American Physiological Society.

**Figure 2:**
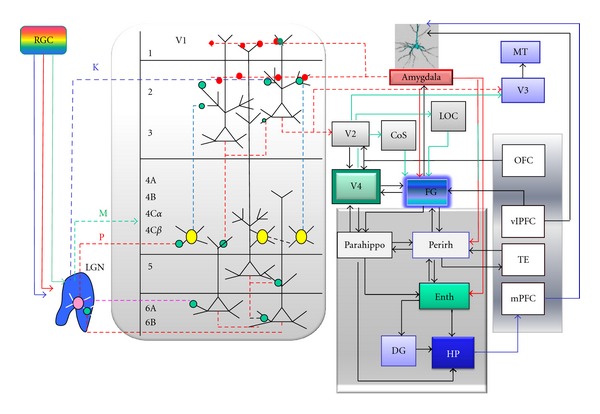
Visual signal processing along the ventral visual stream. Photons reflected from the object surface traverse first three retinal cell layers to reach photoreceptor-containing cones and rods. Retinal image formation relies mainly on differential glutamate signalling by ON and OFF cones [19, 40]. Local calculations performed by dendritic branches of direction-selective retinal ganglion cells (RGC) and asymmetric nature of synaptic inhibitory inputs from starburst amacrine cells assure high fidelity of object image formation at the retina [41]. Each pixel of the retinal image gets transmitted via dedicated RGC axons to the lateral geniculate nucleus (LGN). Propagating action potentials excite parvocellular LGN neurons (P), which synapse onto stellate cells of V1 (4C layer, yellow) [19]. Direct koniocellular afferents (K) from LGN to L2/3 inform our brain about the relative retinal image displacement (object movement) and activate the dorsal visual stream targeting the orbitofrontal cortex (OFC) [24]. OFC sends rich cholinergic top-down afferents to visual cortex [42, 43] and helps to maintain attention load exercised on V1–V4 areas. The layer L2/3 neurons of V2 send horizontal axonal projections to V4 area, which serve as visual short-term memory buffer [44]. Early visual cortex communicates with fusiform gyrus (FG) and amygdala nuclei of both hemispheres. Primate amygdala projects axons equipped with bouton terminals (dotted red lines and circles) onto dendritic spines located in L1 and L2 layers of V1 [45]. Amygdala-induced neurotransmitter release at the axo-spinous synaptic contacts visual brain areas facilitates formation of visual long-term memories, especially those of high emotional dimension. CoS—collateral sulcus, LOC—lateral occipital cortex, Perirh—perirhinal cortex, Enth—entorhinal cortex, Parahippo—parahippocampal gyrus, DG—dentate gyrus, Hp—hippocampus, vlPFC—ventro-lateral prefrontal cortex, mPFC—medial prefrontal cortex, and TE—temporal lobe.

 A majority of the outside-world stimuli reach our brain via visual and auditory channels. A photoreceptor layer of each retina captures an instant snap shot of the scene, which is then transmitted, filtered, and reproduced by means of the electrical activity of neurons in the visual cortex [18, 19] (Figure 1). The activity of the occipital cortex is modulated by several sensory, associative, attention- and language-devoted brain areas [20]. One question about the visual long-term memory (LTM) storage needs to be resolved. Is the latter assured by the visual cortex or, instead, our mental imagery is stored in a semantic form? One may argue that the crows got excellent visual memory for the human faces (beyond five years) [21] and colours [22] without having developed the lingual brain. The evidence that the visual LTMs associated with human faces are maintained by the neural networks between the fusiform face area (FFA) [23] and the fusiform gyrus (labelled FG in Figure 1) comes from patients with posterior cerebral artery strokes [24]. The latter is usually manifested by loss of the human face recognition and sometimes by the object category-specific agnosia. How do we recognize familiar faces, or objects? Different neuron types arranged in six layers of the primary visual cortex V1 (Figure 2) initially transmit the information about their shape, colour, orientation, and movement to dedicated brain areas along the ventral [18] and dorsal [25] visual streams. The visual field mapping by V1–V4 [26, 27] is accompanied by the shape analysis in the lateral occipital cortex (LOC), colour in the anterior collateral sulcus (CoS)/lingual gyrus, and texture in the posterior CoS area [28] (Figure 2). While V1 and V2 (mainly Brodmann areas 17 and 18) are sensitive to reflectance properties of perceived surfaces [29], distinct anatomical locations of the right FG respond to object categories such as human faces, body parts, animals, houses, and man-made tools [30–33]. These make sense of subtle shape-colour-texture differences between objects composing the scene [27, 28]. The left FG has been proposed as multimodal (visual, tactile, and auditory) memory storage site for everyday manipulable objects [34]. Rossion and colleagues [33] have recently studied time-dependent recognition of human faces versus cars. The authors have identified direct sensory inputs from the early visual cortex to the right middle FG (right fusiform face area, or FFA) by BOLD fMRI. Actually, the right FFA gets activated before the left LOC, which seems to depend very little on the inferior occipital cortex [33]. On the other hand, the left middle FG contains the so-called visual word form area (VWFA) and responds to letters, words [35] and tactile reading of Braille symbols (visual experience-independent process in congenitally blind people) [36]. The left VWFA has been suggested to assist retrieval of lingual object representations from the left lateral temporal lobe [37]. The lingual-type memory retrieval upon visual object perception is usually so rapid that we feel the object name by the tip of our tongue in a fraction of a second.

Interestingly, the written words FACE or HOUSE, giving an indication about an image category to be presented, enhanced selectively BOLD response in the left FFA, or parahippocampal area [46]. The anterior part of FG sends axonal projections to the perirhinal area, which communicates via the lateral entorhinal cortex with the hippocampus [39] (Figure 2). The visual scene boundary mapping by the hippocampus [47], parahippocampal area, and LOC [48] seems to be of critical importance for our instant comprehension of the scene and for navigation in a complex environment.

The present paper focuses on the molecular biology of visual perception in human as compared to that of rodents (Sections 2 and 3). It points at multisensory perception of broken objects by humans (Section 4) and discusses how the amygdala facilitates conversion of sensory-type short-term memories (STM) into relatively stable LTM (Sections 5 and 6). The main goal of the paper is to identify possible mechanisms of the visual long-term memory formation and maintenance across the human brain.

## 2. The Primary Visual Cortex at Work

Visual object perception starts with the formation of the retinal image and its transfer from the retina as parallel electrical signals (action potentials) by the optical tract to the lateral geniculate nucleus (LGN) (Figures 1(a) and 2). In order to reproduce object contours by the primary visual cortex V1, the parvocellular and magnocellular LGN neurons must excite the spiny stellate cells in the layer 4C*β* [19] and pyramidal neurons of layer 6 [49] (Figure 2). Stellate cells project to layers 2 and 3 (L2/3) of V1, where a visual stimulus induces glutamate release at their axon terminals. Released neurotransmitter binds to dendritic spines of L2/3 pyramidal neurons and opens up their N-methyl-*d*-aspartate receptors (NMDAR) and AMPAR, which causes ion influx into the cell. The overall spiking profile of a given L2/3 pyramidal neuron depends on the extent of its targeting by other neurons expressing GABAergic, cholinergic, adrenergic, and other receptor subtypes [50, 51].

A drop in the neuron firing is constantly readjusted by insertion/removal of AMPAR [52–56] and NMDAR depending on the distance from the soma (synaptic scaling) [57, 58]. Most of the isolated spike signals arriving from LGN potentiate dendritic spines in V1 only weakly, however, certain spines experience repetitive trains of high-frequency electric stimulation (>20 Hz), accompanied by Ca^2+^ entry via NMDAR [59]. This gives the signal to up-regulate AMPAR incorporation at the postsynaptic membrane in the process called the long-term potentiation (LTP) [60, 61]. In naive spines, LTP requires activation of several AMPARs and strong enough depolarization of the postsynaptic membrane to release Mg^2+^ and unlock the NMDAR [62]. On the other hand, the low-frequency electric stimulation (<10 Hz) induces a more massive Ca^2+^ entry into the spine and results in AMPAR internalization, called also long-term depression (LTD) [62, 63]. LTP and LTD are expressed unequally from the apical tuft towards basal dendrites of the pyramidal neuron, and across the cortical layers from L1 to L6, due to the differential distribution of NMDAR, AMPAR, metabotropic glutamate receptors (mGluR), and various ion pumps [64, 65]. The LTP may be induced at several spines at once providing these reside on the same dendritic branch [66].

The Rho GTPase family kinases (signalization network downstream of NMDAR and AMPAR) communicate with the calcium-dependent kinase CAMKII and are able to spread laterally tens of microns away from the original LTP site [67–69]. Importantly, LTP may be induced by diffusing enzymes even in certain silent spines [66]. The entire dendritic branch is then able to memorize the stimulus direction for short or extended periods of time depending on spine types and their local protein machinery [66]. It has been observed that the synaptic connectivity between neighbouring L2/3 neurons with the same orientation preference progressively strengthens (stronger synaptic contacts being formed) as compared to that with uncorrelated responses [69]. Such orientation-selectivity of dendrites gets rapidly translated into oscillations of the somatic membrane potentials and firing pattern of the parent pyramidal neuron [70, 71]. Membrane oscillations of individual neurons may in turn get synchronized with other neurons having the same orientation preference. The arrangement of orientation-sensitive domains across the primary visual cortex has a beautiful patch-like appearance (see Figures 1 and 2 in [72]) possibly reflecting the Moiré interference of the hexagonally arranged ON- and OFF-retinal ganglion cells [72].

The information transfer across the visual cortex takes place mainly at synaptic contacts between the axon terminals and spines equipped with glutamate receptors. Kwon and Sabatini [73] have recently answered several fundamental questions regarding the kinetics of dendritic spine formation at rodent L2/3 neurons. The authors have shown that the local application of glutamate at 10–12 day sold L2/3 neurons results in the appearance of new spines within seconds! This required calcium entry via NMDAR followed by cAMP-dependent PKA activation.

In contrast to that, the spine enlargement in older animals (>20 days) required the electric activity-dependent LTP, the TrkB receptor activation by BDNF and downstream MAPK- and CAMKII signalization pathways.

## 3. Object Contour Analysis by V1 Neurons

Zilberter and colleagues [59] studied the precise locations of synaptic contacts and signal transmission between neighbouring pyramidal neurons in rat V1. The synapses, formed between L2/3 presynaptic axonal boutons and postsynaptic sites, were detected mainly on proximal basal dendrites. An action potential burst at 10 Hz induced LTD at L2/3 pyramidal cell-pyramidal cell connections, while an increase in the burst frequency to 20 Hz switched LTD to NMDAR-mediated LTP. The latter phenomenon seems to follow the logic of membrane-potential based rules [74] summarized recently by Spruston and Cang [75]: “When neurons A and B are activated together at rates greater than about 10 Hz, both the LTP and LTD conditions are met, but the LTP is larger, so strong bidirectional connections develop”. Spike timing-dependent synaptic plasticity (STDP) [76–78], that is, time-dependent synchronization between the presynaptic glutamate release site and the postsynapse, decides whether a given synaptic contact will be strengthened (tLTP) or weakened (tLTD) [79] (for a recent review, see the paper by Larsen et al.) [78]. Feasibility of the tLTP or tLTD induction in striate cortex varies during the brain development and depends on the NR2B/NR1 ratio (NMDAR composition), the presynaptic signalization network downstream of the cannabinoid CB_1_R receptor (L2/3 of V1), and other factors [80]. The excitatory glutamatergic network rapidly adapts to dramatic morphological changes occurring during the maturation of the local GABAergic network (see Figure 6 in [81]), and *vice versa *[82].

Formation of the retinotropic map in early visual brain areas requires both excitatory and inhibitory circuits. The excitatory signal propagates preferentially along certain dendrites of direction-sensitive L2/3 pyramidal neurons, while other dendritic branches are kept under the inhibitory control of somatostatin-(SOM-), parvalbumin-(PV-), and calretinin-(CR-) positive GABAergic interneurons [81, 83]. In primates, collective oscillations of L2/3 pyramidal cells with soma-targeting PV-positive interneurons (gamma-band oscillations) have been suggested to be responsible for the orientation-selectivity of the V1 area [70, 84]. On the other hand, L2/3 pyramidal neuron targeting by SOM-positive- (rather than PV-positive) GABAergic interneurons assure orientation selectivity in the striate cortex of rodents [85]. The primate L2/3 pyramidal neurons target L5 and L6 neurons of V1, but send also horizontal axon projections to V2 [86] and transmit the excitatory electric signal to deeper visual brain areas. The L6 neurons of V1 back-project to LGN (see Figure 2). Even though the excitatory signal sent by primate retina reaches first V1, the voltage-sensitive dye imaging (VSDI) pattern changes at the single-pixel level indicate that V1, V2, and V4 have already worked simultaneously as early as 40 ms following the image perception (see supplementary Figure S6 in [87]). In the case of images of emotional nature, the membrane oscillations of GABAergic and dopaminergic interneurons of the limbic circuit including amygdala may get synchronized with neurons of the visual cortex (Figure 2). Osipova and colleagues [87] suggest that such collective oscillations of neuronal assemblies in the gamma frequency range in V1/V2 may be associated with the memory encoding/retrieval coupled to mnemonic operations in the theta range (4–8 Hz) across the right parietotemporal areas. In rodents, the reward-associated dopamine release in the ventral tegmental area (VTA) locks the oscillations at 4 Hz and synchronizes VTA with the hippocampus and the medial prefrontal cortex (mPFC) [88].

## 4. Multimodal Sensory Perception of Broken Objects by Humans

Perception of the broken coffee cup by humans has been chosen here since such irregular object shapes appear to be good substrate for the visual rather than the lingual-type of LTM encoding. Humans are simply missing words to describe the exact shape of the missing fragment. From an fMRI standpoint, the coffee cup belongs to the category of neutral, nonliving, and motionless objects of round shapes. In practice, our visual perception of a white coffee cup does not elicit emotion. However, the same does not apply for a broken coffee cup. The accidental drop of the ceramic object on the floor usually results in its damage and practically instantaneous expression of emotions.

In infants, the initial moment of surprise shifts to the sudden feeling of joy and irresistible desire to break another object. In older children, the feeling guilt and fear mix together and may lead to the diverse strategies and attempts to hide the accident. In adults, the coffee cup breaking is often accompanied by swearing, which tends to calm down our initial excitement and anger. While the human brain is able to efficiently erase visual memories encoding the exact shape of a broken sugar cube we drop in our coffee, we seem to remember the shape of the broken coffee cup even though the accident happened some time ago. We are not likely to forget in what shape our car was following a traffic accident, even though the accident happened many years ago. Thus, it seems the more personal the story gets and the more value we assign to the object, the more efficient are the processes of long-term memory formation, maintenance, and retrieval. It has been noticed only very recently that amygdala activation takes place not only during intense emotional events, but also when evaluating an object's value [89]. Interestingly, the amygdala sends numerous axonal connections to several brain areas and, due to noradrenaline release [90], it contributes directly to the robustness of LTM formation and maintenance [91–94].

At first glance, our visual perception of the coffee cup and its broken counterpart should be rather similar. The only difference resides in the exact shape of the missing fragment. Our brain must capture such contour differences quite early during the retinotropic map formation in early visual areas. Once the fragment contours captured by V1–V4, other visual brain areas responsible for the stereoscopic depth processing and analysis of complex 3D shapes need to be recruited [95]. The left fusiform gyrus has been suggested as some kind of storage site for the long-term trisensory representations associated with manipulable objects [46]. For example, in the case of the coffee cup accident, this could cover the shape of the broken object, auditory memories associated with an object falling on the floor and tactile sensations experienced while collecting the pieces of the broken ceramics. Whether FG is indeed a multimodal LTM storage site or, instead, separate memory stores exist for specific object-sound associations remains to be determined. In this respect, a recent study using diffusion tensor imaging (DTI) combined with fMRI [96] traced down independent storage sites for visual- and auditory-type LTMs linking a specific human face to a human voice. While face perception activated face-selective FG, the voice belonging to the person activated systematically the superior temporal sulcus (STS). The proper name “coffee cup” is likely to activate the anterior temporal lobe (ATL). In that context, the case of a salesman in a kitchenware store is very interesting. He could recognize objects, but forgot how to name certain kitchen utensils he had sold before the surgical left ATL dissection [97].

## 5. Effects of Amygdala on Memory Encoding

Let us take a look at how the memories of the broken coffee cup could have been formed and shaped from the first seconds to several hours following the accident. Initially, the visual cortex keeps the sensory information about the broken object contours in its short-term visual memory system [27]. Multiple rounds of excitatory waves across the primary and secondary visual cortex strongly potentiate neurons in deeper visual brain areas [98]. Sustained brain activity in the theta frequency (4–8 Hz) [99–102] coupled to gamma frequencies (40–80 Hz) [103] might render certain pyramidal neurons in deeper visual areas sensitive to the broken cup contour. This is likely to be facilitated by initial expression of emotions and object value evaluation by the amygdala [89, 93, 94]. The amygdala heavily projects to the orbitofrontal and the prefrontal cortex in primates [104], but equally to the secondary and primary visual areas [91–94]. Norepinephrine release from terminal axonal boutons is known to modulate the horizontal cortico-cortical signal transmission along L2/3 neurons [105] by activating *β*-adrenergic receptors. Norepinephrine binding to *β*_2_-adrenoreceptor causes release of the G_s_ subunit from a GPCR. This activates adenylate cyclase, which generates locally high levels of cAMP and recruits *β*-arrestin. The latter protein binds also to the *β*_2_-adrenoreceptor and activates B-Raf and ERK signalling pathways [106]. In animal models, the norepinephrine release directly affects local protein synthesis in spines required for memory consolidation and storage beyond 3 hours following the LTP induction [107, 108]. Emotion-induced dopamine and norepinephrine release helps to convert early-LTP into late-LTP [94, 108–111]. The late-LTP, which can last weeks and longer, is usually thought of as the physical substrate underlying LTM. Hippocampal day replay [112] and night replay [113–115] of the scene could lead to reinstatement of hippocampus-driven memories. Interestingly, the intra-hippocampal injection of the brain-derived neurotrophic factor (BDNF) in rat increases ryanodine receptor (RyR2 and RyR3) as well as protein kinase PKM*ζ* expression levels [116]. This fact points at the important role of calcium-induced calcium release (CICR) via ryanodine receptors in the late phase of the LTM formation.

Independently from the exact location of the LTM storage site, neuron clusters and individual neurons sensitive to our broken coffee cup shape are unique in the sense that they are not linked to any other event, place, or time in our life. The fact that the human brain is able to locate such specific LTM-keeping clusters among millions of others, retrieve object-place associations, activate the amygdala and instantly express emotions is quite remarkable. Emotions are expressed instantly following the visual perception of the broken object and memories retrieved in a fraction of the second even years later. These observations reflect the fact that related neural networks were formed at the same time, are wired together and, therefore, reactivated together.

Let us consider what separates neuron populations encoding the broken coffee cup shape compared to those keeping, for example, our credit card number. The five- or six-digit numeric code is usually learned within a day or two and related LTM reactivated almost daily during four year credit card period. On the other hand, remembering the shape of the broken coffee cup seems to be effortless, and related LTM may be retrieved sometimes years later without daily memory training. It is likely that the six-digit code-encoding neuron cluster would be either lost or inaccessible for LTM retrieval following the same time period. A simple explanation of the phenomenon could be that our brain allocates very little resources to storage of numeric representations in the intraparietal cortex [119]. Eventually, such neuron clusters are smaller in size (or less numerous) than those encoding visual LTMs. The human visual memory seems to have a massive storage capacity for object details (close to 90% accuracy for 2500 objects viewed during 5.5 hours) [120] but negative emotions seem to worsen memory accuracy [121]. On the other hand, the random numbers rarely induce any particular emotion. As a result, these might not couple strongly to fear- or reward- activated brain regions.

BOX1: Forms of visual memory. The vision science makes the distinction between the iconic memory (<500 ms), visual short-term memory (vSTM), and visual long-term memory (vLTM). Slighte and colleagues [44] have shown that the fMRI BOLD activity in the V4 area may persist upon retention of a dozen of objects in the early visual cortex up to four seconds [44]. This process is subject to top-down attention control from the posterior parietal cortex and frontal eye fields [42–44]. An iconic memory and weak vSTM seem to be unstable in respect to subsequent visual stimuli, while a strong vSTM (high attention load) can survive for extended periods of time [44]. It is well known that the success of visual memory retrieval degrades rapidly with increasing number of objects (the scene clutter) and/or increase in complexity of object shapes [122]. This has been explained as due to difficulties with task switching between frontal lobe areas and the posterior parietal cortex when trying to retrieve colour, shape, and form of perceived objects [123], eventually, their lingual representations [124]. According to Brady et al. [122], the vLTM has a rather low storage capacity as compared to “stored visual knowledge.” The latter might facilitate object feature extraction and retinotropic map decoding by activating visual, lingual, and frontal brain areas. Such processes are likely to be multitask switch-dependent and activate the hippocampal formation, fusiform gyri, and frontal and temporal lobes. The retrieval of existing vLTMs is expected to rely on the local sensory stores. What is the minimum time requirement for the visual LTM formation? Lewis and colleagues [125] studied the speed and accuracy of mental image generation for the arrangement of 2–8 black dots on a grid. The vSTM task lasted 5 seconds, while the visual long-term memory paradigm about 800 seconds (ca. 13 min). From molecular biology point of view, it is clear that the neural networks keep such newly acquired information in the early-LTP-based system (extending beyond the visual cortex) rather than LTM. The LTM formation associated with the perceived image would require at least 2-3 hours following its acquisition (memorization). The neural networks implicated in the cognitive process need several hours in order to strengthen their synaptic contacts and synthesize new proteins needed for LTM formation/maintenance (see below).

## 6. Visual LTM Maintenance and Retrieval

**Figure 3:**
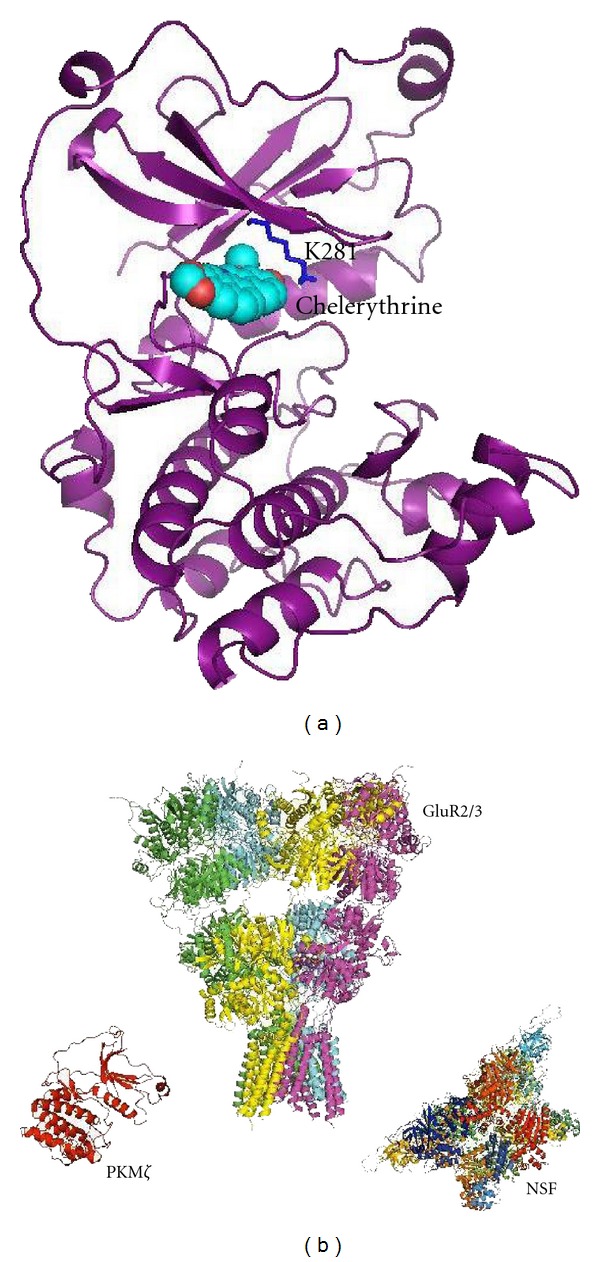
3D models of proteins involved in the long-term memory maintenance: (a) human PKM*ζ* homology model based on PKC iota [1ZRZ.pdb]. The chelerythrine (sphere-filled model with the oxygen atoms shown as red spheres and carbon atoms as cyan) binding site was identified within the ATP-binding pocket of the PKM*ζ* (ribbons). Docking was performed using AutoDock software [117] and protein was visualized using PyMol. The inhibitor binding site is located in the vicinity of the Lys281 residue. K281W mutation renders PKM*ζ* inactive and causes permanent erasure of the long-term memory [118]. (b) The N-ethylmaleimide-sensitive factor (NSF) consists out of two hexameric Walker-type D1 and D2 domains (N-terminal NSF domain not shown). Its ATPase-catalytic activity is needed to release GluR2 subunit from endosomes and GluR2/3 assembly at the postsynaptic membrane. Crystallographic structure for the last 68 amino acids of the GluR2 C-terminal, including the NSF and AP2 binding sites, is not available.

Where do we store visual long-term memories marked by emotions? Sacco and Sacchetti [126] have recently provided experimental evidence that emotionally enhanced visual, auditory, and olfactory LTMs depend on the atypical kinase PKM*ζ* (Figure 3) and are stored directly in the secondary sensory areas. More specifically, rat visual LTMs, stored in the temporal lobe (TE area), were shown to be permanently erased by local application of the *myr*-zip peptide (myristoylated PKM*ζ* inhibitor).

Zip-peptide, the lysine 281 mutation to tryptophan (K281W) in PKM*ζ* and the injection of alkaloid chelerythrine (Figure 3) into LTM-keeping brain areas can permanently erase long-term memories [118, 126–132]. Emotions may induce neurotrophin (BDNF and NGF) release directly in the hippocampus, amygdala, and also in amygdala-projecting areas including the visual cortex [133, 134]. Dramatic changes in the volume of certain spines take place in minutes following the LTP induction [133]. BDNF binding to TrkB receptors activates the MAPK/ERK kinase pathway, which induces early gene expression (CREB, Arc/Arc3.1, zif268) [135, 136]. Alternation between neuron oscillations in the theta frequency (4–8 Hz) [102] and gamma frequency (40–80 Hz) [103] range may be responsible for switching between the LTP and LTD, which serves to synchronize the complex transcription/translation machinery within the spine [94]. Memory formation requires hundreds of proteins, produced locally in the spine and dendrites, to assure the spine growth and maturation. This includes actin branching and stabilization by actin-binding proteins and their phosphorylation by CAMKII [137], CdC42, RhoA, and other kinases [67–69]. However, during the first two hours of such accelerated spine changes, the protein kinase PKM*ζ* is absent from the scene possibly due to the translational block imposed by the prolyl isomerase PIN-1 [138]. A second wave of BDNF release (>3 hours following the LTP induction) signals axon-located mitochondria to produce more ATP [139]. Axon guidance proteins and associated kinases promote axon outgrowth, axon branching and increase the number of axon terminals within the newly formed memory cluster [110]. The BDNF signalization network seems to protect the atypical kinase PKM*ζ* from its degradation in local proteasomes [140]. On the other hand, the kinase PKM*ζ* selectively phosphorylates the zinc finger protein ZDHHC8 responsible for the PSD95 palmitoylation and its targeting to synapses [141]. Emotions contribute to memory formation not only during such early and late LTP phases, but equally assist the late-LTP/LTM transition within the spine [107, 142]. Electron microscopy has revealed that small and certain large spines disappear within 24 hours, while those marked by LTP (learning) undergo specific changes in the spine volume and structure at the axono/synaptic interface [109]. Such synapse changes and increased protein turnover might occur from several hours to several days. The human emotions might help to connect neurons within the same LTM cluster efficiently together, especially those establishing strong synaptic contacts simultaneously during the same event marked by emotions. Even though the role of atypical kinase PKM*ζ* in LTM-maintenance in several brain areas has been clearly established [132], its implication in LTM storage in visual cortex has been largely ignored. Marc Bear and colleagues [143, 144] were first to demonstrate the important role of PKM*ζ* in rodent vision. Yao and colleagues [129] have suggested that PKM*ζ* may act through NSF to release GluR2-containing receptors from a reserve pool held at extra-synaptic sites by protein interacting with C-kinase 1 (PICK1). Joels and Lamprecht [145] have recently demonstrated that the GluR2-NSF interaction inhibitory peptide (pep-R845A) causes rundown of EPSC in rat lateral amygdala. The inhibitor injection causes AP2-dependent GluR2 internalization and inhibition of fear memory consolidation and retention in the amygdala. The large mushroom-type spines containing calcium-impermeable GluR2/3-type AMPARs are more appropriate for the LTM storage as compared to those containing homomeric-and calcium-permeable AMPAR [135]. GluR2/3 is maintained at the postsynaptic membrane mainly due to the GluR2 C-end binding to ABP/GRIP (PSD adapter proteins). However, the complex between the GluR2 C-end/palmitoylated GRIP is not permanent and AAA^+^ ATPase Thorase is able to disassemble the AMPAR-GRIP1 complex and induce AMPAR endocytosis and LTD [146]. One of ways of maintaining GluR2/3 AMPAR at the postsynaptic membrane is to continuously remove its subunits from endosomes and increase their residence time at the postsynaptic membrane [135]. This seems to be the role of constitutively active protein kinase PKM*ζ* working together with the NSF ATPase (Figure 3). The exact target of the PKM*ζ* at the endosome level is not known, but *β*-SNAP is one of the likely candidates. The latter protein keeps NSF under the inhibitory control [147] and was found expressed at high levels in the primary visual cortex of cats [148]. The relative amount of co-localized GluR2/3 subunits in the primate visual cortex is rather low, but they are clearly present in layers 2/3, 5 and 6 of macaque V1 (practically absent in GABAergic interneurons) [149]. GluR2/3 content increases dramatically then going towards V2, V4, TEO, TE and hippocampal formation (see Figure 3 in [149]). Co-localization of GluR2/3 in the human temporal lobe (Brodmann area 21) by immuno-cytochemical staining revealed also to be positive in layers L2/3, L5 and L6, with very little of staining in L4 [150]. Would low concentrations of *myr*-zip peptide induce endocytosis of postsynaptic GluR2/3 receptors, spine shrinkage and incorporation of calcium-permeable AMPAR (original LTM loss, but the spine survival)? In the opposite scenario, the mushroom-like spine could experience a more serious damage and apoptotic cell death. The spine growth and shrinkage in rat visual cortex has recently been investigated *in vivo* by two-photon imaging microscopy [151]. However, to my knowledge, there is no microscopy study showing synaptic volume changes following the PKM*ζ* inhibitor application in any brain area. Since PKM*ζ* presence in LTM-expressing spines leads to doubling of GluR2/3 receptors at the postsynaptic membrane [135], the immunochemical enzyme co-localization with GluR2/3 receptors could allow identify pyramidal neurons implicated in the LTM storage.

## 7. Conclusions and Future Directions

One of the goals of the 21st century neuroscience will be to understand our vision on a molecular level. Recently available novel technologies and experimental approaches allow studying brain structure-function relationship in animal models. Classical loss-of-function and gain-of-function studies have been extended for the light-controlled neuron activation, the incorporation of non-canonical amino acids into newly synthesized brain proteins [152], or 3D-reconstruction of neurons and spines using neuron array tomography [153]. A realistic image of the letter M [154] and short movies [155] were recently decoded by measuring BOLD activity in V1/V2 areas (Figure 1(b)). The ultimate proof that the BOLD response indeed follows localized changes in the neural activity comes from optogenetics (laser-controlled activation/inhibition of neurons) by means of viral vector-delivered light-sensitive opsins [156]. The high-resolution 9.4 T BOLD fMRI [157] was able to trace down the activity of the light-activated neocortical neurons even in a small rodent brain. Even more exciting is the fact that specific fear memories could be reactivated by shining the laser light on a rather small group of hippocampal neurons (dentate gyrus) of the genetically-modified mouse [158]. The latter experiment suggests that old memories might not be transferred from hippocampus to other brain areas (as believed some years ago) but, instead are induced, driven and reactivated by the same set of neurons physically interconnected with their partners (originally marked by learning) in different brain areas (see also the discussion in the Section 5).

In spite of the exciting developments in neuroscience, as briefly outlined above, a general consensus regarding how the human brain handles memory formation, storage and retrieval is yet to be reached. BOLD fMRI is commonly used to track which brain areas get activated during sound hearing or picture viewing. However, it remains to be understood where resulting LTMs are stored. Even though the GluR2/3 receptors are less abundant (ca. 10–15%) than GluR1/2 and GluR1 homomers in the rat hippocampal CA1 neurons [159], it is likely that their counts will be much higher in the human brain. The primate brain receives rich axonal afferents from the amygdala which, together with dopamine release induced by VTA, facilitates GluR2-containing receptor expression during LTM formation and maintenance. The co-localization of PKM*ζ*, GluR2/3 and postsynaptic NSF using high-resolution neuron array tomography in post-mortem human brain tissues would be of great interest. Equally, it will be important to identify AMPA receptor subtypes involved in the LTM maintenance across the human mesolimbic reward circuit. In this regard, it is interesting that the injection of two PKM*ζ* inhibitors (chelerythrine, or *myr*-zip peptide) within the nucleus accumbens core [160] and VTA [161] blocked place preference LTMs associated with cocaine reward in rodents.

Very little is known about the distribution of the ionotropic and metabotropic glutamate receptors in the human fusiform gyri. This concerns the AMPAR subunit composition, postsynaptic-density proteins and enzymes involved in the LTM formation and maintenance. The surgical dissection of the left FG resulted only in difficulties with orthographic processing such as retrieval of word spelling from its meaning in writing (but practically no alteration in visual perception of faces and objects) [162]. On the other hand, the damage to the right FG resulted in altered face recognition. The damage to the right FG and LO area resulted in face and object agnosia [163]. This is in line with earlier BOLD fMRI studies by Grill-Spector and colleagues [164] who have concluded that the right—rather than left FG handles categorization of faces, limbs, tools or animals. Recently, the first case of a developmental deficit in object recognition but normal face recognition in a young female patient has been reported [165]. The authors explained that the associative form of congenital prosopagnosia (hereditary disease) was due to the damaged white-matter tract between the occipital face area (OFA) and FFA (right hemisphere) [166]. Indeed, the existence of white matter fibers linking OFA and FFA has recently been demonstrated using the DTI tractography [167]. Therefore, it seems that the right FG stands out as the important visual memory processing/storage site, while the left FG functions as complex interface between the visual and lingual brain.

## 8. Glossary

### 8.1. Synaptic Plasticity

Changes in the composition of membrane receptors and membrane proteins affecting communication between the excitatory or inhibitory neurons.Many of plasticity-related phenomena rely on calcium-permeable glutamate receptors and Ca^2+^-dependent signalization network downstream of NMDA, AMPA and mGluR receptors.

### 8.2. Memory Formation

At the single neuron level, changes in the spine volume and protein composition due to frequency- or chemical-dependent forms of learning such as LTP or LTD. Long-term memory formation usually requires alternation of LTP and LTD in order to switch between the transcription and translation machinery steps needed for the local protein synthesis within the spine.

### 8.3. Dendritic Spine

 A small protrusion on a dendrite surface equipped with membrane receptors and hundreds of proteins and enzymes. Spine volume and shape often indicate whether it is implicated in neuron-to-neuron communication (small spines), eventually, the memory storage (mushroom-like spines).

### 8.4. Synaptic Scaling

 In order to maintain neuron firing profile imposed by the neural network, the number of glutamate receptors, their subunit composition, and phosphorylation pattern change depending on the spine location on the dendrite in respect to the action-potential generating somatic cell compartment.

### 8.5. Spine-to-Spine Signalization

In spite of important degree of compartmentalization and specialization, the spine is far from being a closed system. It communicates very rapidly by means of diffusing kinases with other spines residing on the same dendritic branch. This way the spine reports about the progress of its synaptic learning. Similarly, spines communicate with the nucleus even though on somewhat longer time scale (hours). This usually takes place when delivery of specialized cargo proteins is required and such proteins are not available locally or might not be possible to synthesize within the spine.

### 8.6. BOLD fMRI

Blood Oxygenation Level-Dependent functional Magnetic Resonance imagingfollows local changes in the blood flow and local concentration gradient of the paramagnetic deoxy-hemoglobin molecule.

### 8.7. Retinotropic Map

 Point-to-point topography of the retinal image reconstructed by electrical activity of neurons in anatomically distinct visual cortex areas.Visual maps may be conveniently measured by high-resolution BOLD fMRI.

### 8.8. Loss**  **and**  **Gain-of-Function Studies

Genetic manipulations in animal models resulting in deletion of the gene of interest or, on the other hand, introduce novel gene belonging to another organism. Optogenetic gain-of-function studies rely on introduction of genes encoding ion pump proteins from the opsin family sensitive to certain wavelengths in the visible range.
